# The Extrusion and SPS of Zirconium–Copper Powders and Studies of Selected Mechanical Properties

**DOI:** 10.3390/ma14133560

**Published:** 2021-06-25

**Authors:** Tomasz Skrzekut, Grzegorz Boczkal, Adam Zwoliński, Piotr Noga, Lucyna Jaworska, Paweł Pałka, Marcin Podsiadło

**Affiliations:** 1 Department of Material Science and Engineering of Non-Ferrous Metals, Faculty of Non-Ferrous Metals, AGH University of Science and Technology, A. Mickiewicza Av. 30, 30-059 Krakow, Poland; gboczkal@agh.edu.pl (G.B.); zwolo@agh.edu.pl (A.Z.); pionoga@agh.edu.pl (P.N.); ljaw@agh.edu.pl (L.J.); pawel.palka@agh.edu.pl (P.P.); 2 Łukasiewicz—Krakow Institute of Technology, Zakopianska St. 73, 30-418 Krakow, Poland; marcin.podsiadlo@kit.lukasiewicz.gov.pl

**Keywords:** Zr-Cu mixtures, zirconium powders, SPS, microstructures, SEM, mechanical properties

## Abstract

Zr-2.5Cu and Zr-10Cu powder mixtures were consolidated in the extrusion process and using the spark plasma sintering technique. In these studies, material tests were carried out in the fields of phase composition, microstructure, hardness and tensile strength for Zr-Cu materials at room temperature (RT) and 400 °C. Fractography analysis of materials at room temperature and 400 °C was carried out. The research took into account the anisotropy of the materials obtained in the extrusion process. For the nonequilibrium SPS process, ZrCu_2_ and Cu_10_Zr_7_ intermetallic compounds formed in the material at sintering temperature. Extruded materials were composed mainly of α-Zr and ZrCu_2_. The presence of intermetallic compounds affected the reduction in the strength properties of the tested materials. The highest strength value of 205 MPa was obtained for the extruded Zr-2.5Cu, for which the samples were cut in the direction of extrusion. For materials with 10 wt.% copper, more participation of the intermetallic phase was formed, which lowered the mechanical properties of the obtained materials. In addition to brittle intermetallic phases, the materials were characterized by residual porosity, which also reduced the strength properties.

## 1. Introduction

Most of the produced zirconium is used in the nuclear industry, while the rest is utilized in the chemical industry and metallurgy [1]. Zirconium is characterized by excellent resistance to corrosive attack in most organic and inorganic acids, salt solutions, strong alkalis and some molten salts. Most components manufactured for power plants (nuclear reactor core materials such as fuel cladding and guide tubes) are produced by melting and hot and/or cold deformation methods [2]. Zirconium becomes brittle due to its affinity for oxygen, nitrogen and hydrogen [3]. Metallic zirconium may contain 1–4.5% of hafnium. For nuclear application, a hafnium separation should be made [4]. This process is expensive, so the hafnium separation process is not performed in the manufacture of the powders.

Zirconium powders can be produced using various methods: electrochemical, reduction, atomization, hydrogenation (hydrogenation/dehydrogenation (HDH)). The HDH method is used most often; however, zirconium powders have an irregular shape [1]. Contamination of zirconium powders by air (more specifically oxygen) and hydrogen is thus a problem during sintering [5]. This contamination causes an increase in tensile strength and hardness but reduces ductility, resulting in crack formation. New zirconium powder processes have been developed over the past few years. Currently, less contaminated powders are produced. Zirconium powder can self-ignite in the air, especially at high temperatures, which significantly complicates the operations accompanying the production processes using these powders. Zirconium powders allow for a wider use of modern sintering methods and extrusion processes of powders [6].

Zirconium is one of the most biocompatible elements available. Zirconium alloys are considered a substitute metal for titanium in implant applications [7,8]. Zr-Cu binary alloys were prepared using the arc-melting process with zirconium strips and oxygen-free copper by Hong et al. [9]. A series of alloys with nominal compositions of Zr-xCu (x = 0, 1, 5, 10, 15 mass% Cu) were prepared. The magnetic susceptibility of Zr-Cu binary alloys is extremely low, approximately 10^−7^. This level is approximately one order less than that of pure Zr and other commercialized metallic biomaterials. Consequently, Zr-Cu binary alloys have the potential to be used as biomaterials with low magnetic characteristics [9]. Copper is important for metabolism, but at higher concentrations, it can lead to poisoning [10,11,12]. Therefore, direct application of Zr-Cu alloys for implants seems impossible. The author’s previous studies have shown the influence of contaminations in the form of oxygen, nitrogen and hydrogen on the parameters of the SPS process and selected properties of the obtained sintered materials. The impurities of zirconium cause brittleness, influencing metal properties such as tensile strength, hardness, and ductility and also increasing surface tension during melt processing [13,14]. Copper has many of the same properties as zirconium, such as being malleable, ductile, thermally conductive and electrically conductive. Adding zirconium can strengthen copper and prevent deformations. Commercial copper alloys with a 13% to 67% addition of zirconium are produced [15]. Depending on the proportion of zircon to copper in alloys, the purpose of zirconium introduction changes from improving mechanical properties through the phenomenon of strengthening to increasing resistance to corrosive agents. The beneficial effects of zirconium additives in copper are known, but there are individual publications for zirconium alloys with lower copper contents. In this paper, research was carried out on the consolidation of mixtures containing 2.5 wt.% and 10 wt.% Cu by two methods. The first method was spark plasma sintering [16,17,18,19]; the second method was the direct extrusion process. Zirconium is very plastic [20] and has a hardness similar to that of copper; therefore, extrusion tests for Zr-Cu mixtures were also taken into consideration. Previously, extrusion methods were already used for the consolidation of amorphous powders of zirconium materials, such as gas-atomized amorphous Zr58.5Nb2.8Cu15.6Ni12.8Al10.3 [21]. Zirconium alloy extrusion methods are widely used in the production of pipes and other components needed in nuclear power, but in this case, the starting alloys for the commercial extrusion process were castings. For example, the cladding tubes are fabricated from tube-reduced extrusions (TREX) using the Pilger process [22,23]. The main problem impeding the prepared zirconium mixtures and carrying out the extrusion process is the flammability of zirconium powders [24].

In this study, material tests were carried out in the fields of phase composition, microstructure, hardness and tensile strength for Zr-Cu materials obtained by the SPS and extrusion methods. Most components intended for nuclear applications can operate at temperatures above 400 °C (under superheated steam conditions). By increasing the temperature of zircon, in a brittle material, it becomes more malleable. Another effect of heating zirconium is that it becomes chemically active and reacts with, for example, halogens or nitrogen [25].

## 2. Materials and Methods

Commercially procured, high-purity zirconium (supplier Bimotech, Wrocław, Poland) and copper (supplier Kamb, Warsaw, Poland) powders were used The characteristics of purity and particle sizes of these powders are presented in Table 1.

Very early strength tests of two-component and three-component zirconium alloys, including the Zr-Cu system, in order to obtain good mechanical properties, indicate the introduction of metals (Nb, Ta, Ti, Mo, Al, Sn) into the zirconium in an amount ranging from 2% to 10% by weight [25]. Taking into account the limited literature information about strength tests for zirconium alloys and the possibility of comparing the microstructures of Zr-Cu melted alloys [9] to sintered and extruded materials, mixtures of 2.5% copper and 10% copper participations were prepared. According to the Cu-Zr phase equilibrium system for this range of the copper content according to the phase system, the material should contain α-Zr and a CuZr_2_ intermetallic phase [26].

**Figure 1:**
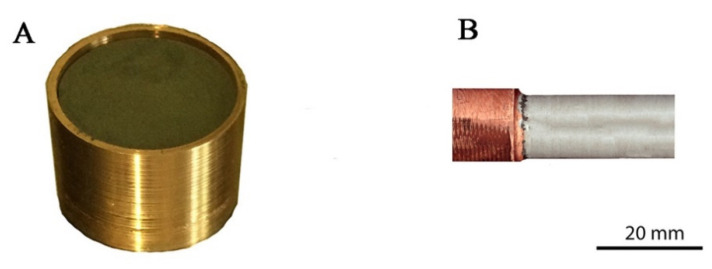
The container with the Zr-Cu mixture before and after the extrusion process: (**A**) container made of Cu-ETP with the Zr-2.5Cu mixture; (**B**) product after the extrusion.

Powders were mixed in a Fritsch Pulverisette 7 (FRITSCH GmbH, Idar-Oberstein, Germany) ball mill with zirconia grinding bowls (size = 80 mL) and grinding balls (10 mm in diameter). The powders were mixed for 16 h in water with a mixing speed of 100 rpm. Despite temperature control, an explosive reaction could occur during mixing, and there was a risk of damaging the bowl. In one of our experiments at a speed of 400 rpm, an uncontrolled explosion occurred due to the large absorption of gases inside the container (hydrogen, oxygen and nitrogen, which were detected in the study of powder contamination in one of our publications [5]), and after this incident, we gave up from the high-speed mixing of mixtures containing zirconium powder. The conditions for the preparation of mixtures with zirconium were developed in the research presented in [24]. The homogenization processes were carried out in the water, in which the zirconium is stored, also. Water is used to protect the zirconium, especially against the absorption of gases from the environment and to reduce its flammability. The mixes for the sintering and extrusion processes were prepared in the same manner. After drying in vacuum, 65 g of the mixture was poured into a container (Figure 1A) made of Cu-ETP (Cu-ETP, CW004A, 99.9% purity; it is produced in electrolytic refining of melted cathode copper). The outer diameter of the container was 39 mm, the height was 26 mm and the wall thickness was 1.8 mm. The container was closed tightly by pressing a specially prepared Cu-ETP plug (Figure 1A). The test of the extrusion process without a container resulted in the ignition of the zirconium mixture. Pressing was performed on a hydraulic press with a maximum pressure of 100 t (Hydromet, Bytom, Poland). The concurrent classic extrusion method was used. The heat treatment, before the extrusion process, was applied due to the strengthening of the material, which results from structural deformation in the mixing process. The consolidation of powders by extrusion was impossible without heating. The powders were placed in a copper container and annealed at 400 °C, 600 °C and 850 °C. The annealing temperature was selected experimentally. The selected temperature ensures the consolidation of the powder after the extrusion process. Finally, before extrusion, the mixture inside the copper container was placed in an oven heated to 850 °C and kept at this temperature for 20 min. After removing it from the oven, the container was immediately placed in the vessel of the extrusion press preheated to 400 °C. The speed of the extrusion (punch displacement) was 3.7 mm/s. The batch diameter was 39 mm, and the product diameter was 19 mm (a 19 mm conical die was used for extrusion) (Figure 1B).

The spark plasma sintering (SPS) method was used for sintering the Zr-Cu powders. The mixtures of powders were sintered in an SPS furnace (FCT Systeme GmbH HP-D5/2, Frankenblick, Germany) to form 20 mm discs in a graphite die (covered by hBN). A heating rate of 100 deg/min was used to the temperatures of 800 °C, 850 °C and 900 °C at a pressure of 35 MPa for 60 s in argon. The density of the sinters and the material after extrusion was measured using the Archimedes method. Observations of the microstructure of the zirconium grains were carried out using a JEOL JEM-2010ARP (Oxford Instruments, Abingdon, England) transmission electron microscope. The shape ratio was calculated using ImageJ (Rockville, Bethesda, MD, USA). The circularity was used as the shape factor. The samples for microscopy studies and for mechanical tests were cut using an electro wire BP05d machine (Zakład Automatyki Przemysłowej B.P., Konskie, Poland). WOLCUT 500 wire (Wolco Sp. z.o.o., Lublin, Poland) with a diameter of 0.15 mm was used for cutting (current of 300 mA and voltage of 76 V). PolyFast resin (Struers, Copenhagen, Denmark) was used to include the samples. The prepared samples were ground on abrasive paper graded from 300 to 4000 and then polished with diamond pastes with a thickness of 3 μm and 1 μm on a Roto-Pol-11 polisher from Struers. The final step in the preparation of the test specimens was the application of the OPS polishing agent (colloidal silica suspension for finish polishing) by Struers. The compacted materials were subjected to X-ray diffractometry. The test was performed with a Bruker D8 Advance/Discover X-ray (Bruker, Billerica, MA, USA) diffractometer using a copper cathode lamp. To provide monochromatic radiation, a wave length of λCuKa1 = 1.540598 Å was used. A scan step size of 0.02° was used for the measurement. The PDF-4 crystallographic base was used for diffraction pattern analysis. The following cards were used to determine the phase: Zr: 01-089-3035, ZrCu_2_ 03-065-7783, Cu_10_Zr_7_: 00-047-1028.

Microstructural analysis and chemical composition analysis of the samples by EDS of polished samples were carried out using SEM (Hitachi SU-70, Hitachi High-Technologies Corporation, Tokyo, Japan). A Shimadzu HMV-2T (Shimadzu Corporation, Kyoto, Japan) microhardness tester was used to perform microhardness measurements (a 19.807 N load was applied).

**Figure 2:**
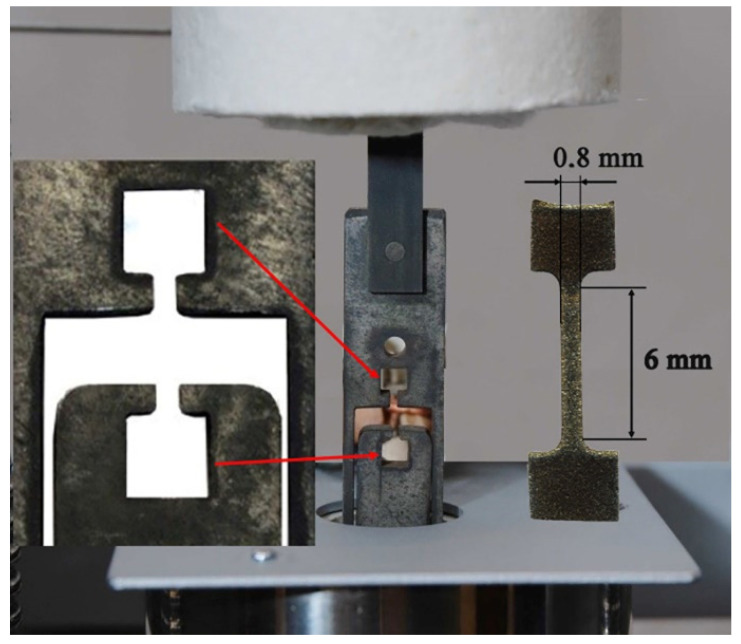
Special holders for the tensile test of the small samples.

The mechanical properties were determined in a tensile test carried out at temperatures of 20 °C and 400 °C. Because of the small size of the samples, they were located in special holders, which were designed for high-temperature tests, to increase the accuracy of averaged values (Figure 2). The dimensions and shape of the samples are shown in Figure 2.

## 3. Results and Discussion

In Table 2, the influence of the temperature of the SPS process on the relative densities and hardness of Zr-Cu materials is presented. The sintering time of the material of 60 s was determined on the basis of minimizing grain growth during the sintering of zircon without additives [5]. The basic criterion for selecting the sintering temperature was the melting point of copper—1083 °C. However, during sintering by the SPS method, which is based on electric discharges between the particles of the sintered material, a much higher temperature may occur at the point of connection of the particles, which often leads to the appearance of a liquid phase at temperatures of at least 100 °C lower than the melting point of the sintered metal, in this case, copper.

The highest values of relative density of about 99% were achieved for both mixtures of Zr-Cu, sintered at a temperature of 900 °C. In Table 3, the influence of the extrusion process on relative densities and values of the hardness of the Zr, Zr-2.5Cu and Zr-10Cu materials are presented. For the extrusion process, the material’s relative density is slightly lower than for sintered materials and amounts to approx. 97%, which is also the case for pure Zr powder. The hardness of the sintered materials is similar to that of the extruded materials (Table 2 and Table 3).

High hardness values were obtained for the materials presented in Table 2 and Table 3. Materials obtained by melting methods, including alloys for nuclear applications, are annealed, which reduces their hardness. For pure zirconium, Vickers hardness HV3 is 145 [27]. The cause of high hardness for sintered materials is thermal stresses. In the authors’ work presented in an earlier publication on the sintering of zirconium, the reduction of thermal stresses (causing the cracking of sintered materials obtained by the SPS method) was achieved by reducing the cooling rate from 100 °C per minute to 50 °C [24]. The oxygen content of the zirconium powder mixtures is 1.6 wt.% [5]. Taking into account the reactivity of zircon, the presence of oxides influences the increase in the hardness of the sintered materials. In the case of the extrusion of zirconium powders, the stresses result from material deformation (strain hardening). The extrusion of zirconium mixtures was carried out at a temperature of 400 °C, which should limit the stresses resulting from material deformation.

**Figure 3:**
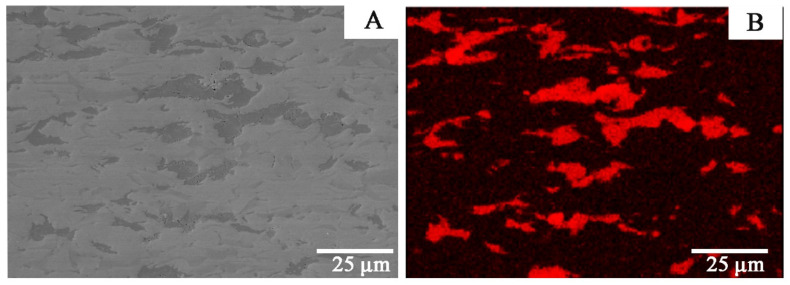
The Zr-2.5Cu microstructure for the material obtained by the extrusion method is presented (longitudinal section): (**A**) SEM microstructure; (**B**) Cu distribution for this microstructure.

**Figure 4:**
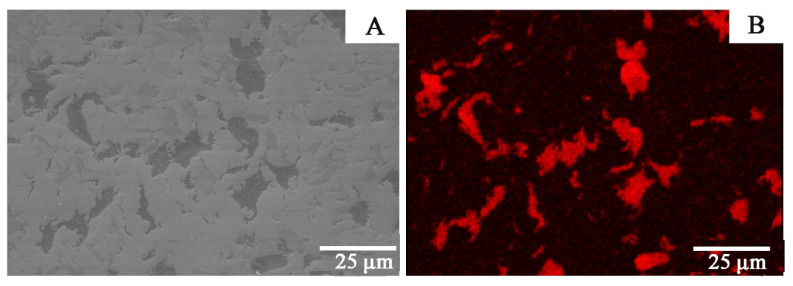
The Zr-2.5Cu microstructure obtained by the extrusion method is presented (cross-section): (**A**) SEM microstructure; (**B**) Cu distribution for this microstructure.

In Figure 3 and Figure 4, Zr-2.5Cu microstructures obtained by the extrusion method are presented.

**Figure 5:**
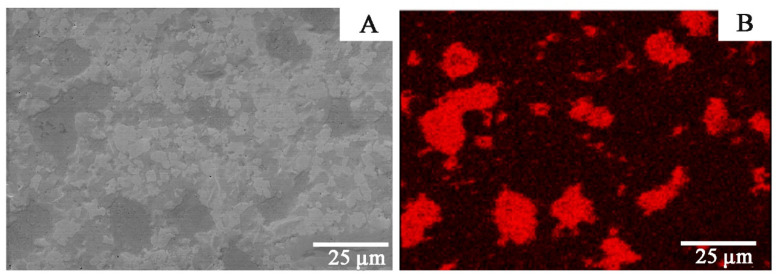
The Zr-2.5Cu microstructure obtained by the sintering (SPS) process is presented: (**A**) SEM microstructure; (**B**) Cu distribution for this microstructure.

Copper accumulates in individual clusters both for materials obtained by the extrusion and SPS methods (Figure 3, Figure 4 and Figure 5). For the material obtained by the extrusion process, the directionality of the microstructure and its elongation in the direction of the extrusion are clearly visible (Figure 3). The microstructure for the cross-section and the longitudinal section of the material obtained by extrusion differs significantly (Figure 3 and Figure 4). For sintered materials, the copper clusters are more isometric in shape (Figure 5). The average circularity for copper grains on the longitudinal section for Zr-2.5Cu is 0.47, and for the cross-section, it is 0.66. In the sintered material, the circularity is 0.81. When the value of the coefficient is equal to 1.0, it means that the analyzed particle is a perfect circle. If the value approaches 0.0, the particle becomes more elongated in shape. The grains in the Zr-Cu sintered microstructures are close to isometric in shape, while the extruded microstructures are elongated.

**Figure 6:**
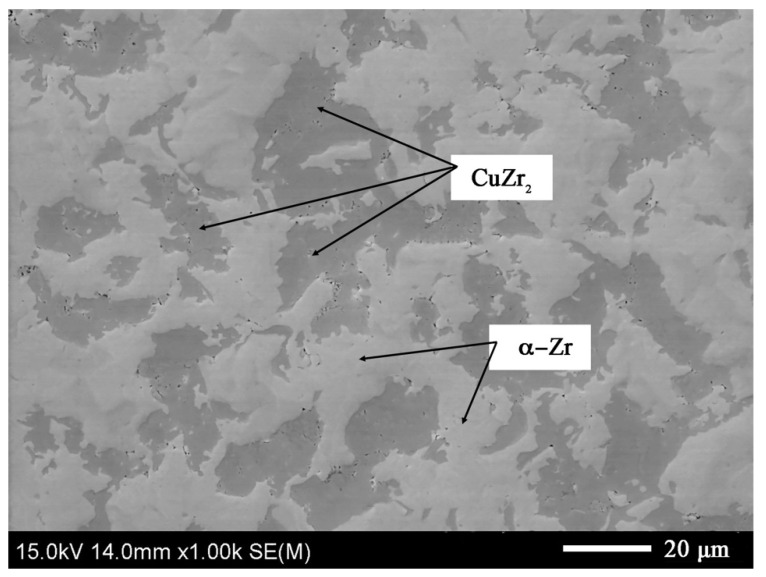
The Zr-10Cu microstructure of the material obtained by the extrusion method: SEM microstructure. Black areas indicate porosity.

The materials are characterized by a high degree of compaction. The micropores in the extruded materials are clustered at the phase boundaries, which are visible in Figure 6.

**Figure 7:**
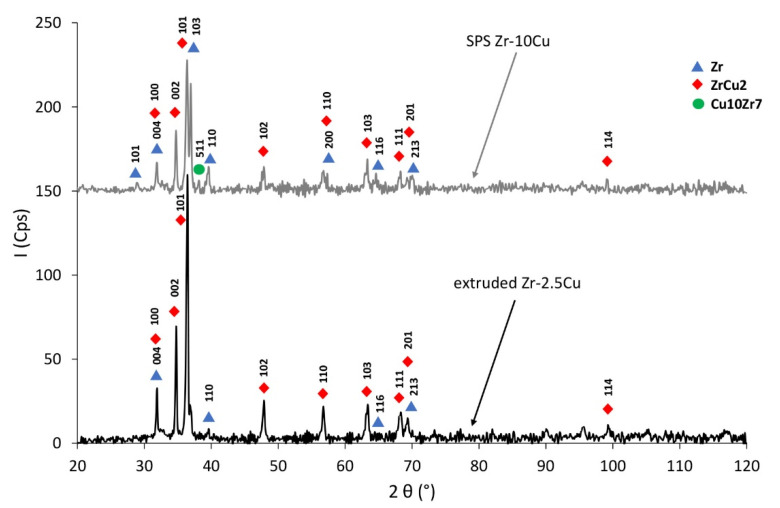
Phase composition of materials: Zr-2.5Cu obtained using the extrusion method and Zr-10Cu obtained with SPS, sintered at 900 °C, 35 MPa, for 1 min in argon.

The Zr-Cu binary alloy for the chemical compositions studied at this temperature range is composed of two phases: α-Zr and a CuZr_2_ intermetallic phase [26]. The dark-gray phases in Figure 3, Figure 4, Figure 5 and Figure 6 are CuZr_2_ grains, which are surrounded by the white α-Zr phase. The microstructures of Zr-Cu sintered, and extruded materials are similar to those obtained by melting methods [9]. The phase composition was confirmed by X-ray diffraction (Figure 7).

X-ray diffractions shown in Figure 7 confirm the presence of α-Zr and ZrCu_2_ (Figure 7). According to the Zr-Cu phase system, α-Zr should occur in this material. For the nonequilibrium SPS process, the ZrCu_2_ and Cu_10_Zr_7_ intermetallic compounds appear in the Zr-10Cu sintered material at process temperature (Figure 7). The Cu_10_Zr_7_ intermetallic phase is formed for the content of 59 at.% Cu, and ZrCu_2_ is formed for the content of 33 at.% Cu [26]. The Cu_10_Zr_7_ intermetallic appears in the sintered Zr-Cu mixtures due to the areas with higher copper content. Copper clusters are visible on the microstructures (Figure 5). The intermetallics of the Zr-Cu system show features of shape-memory materials [28].

**Figure 8:**
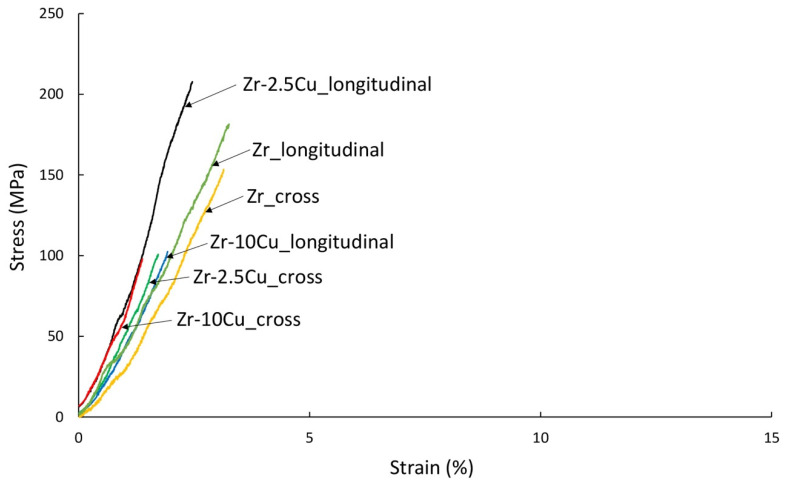
The results of the tensile test of the extruded samples of Zr-2.5Cu and Zr-10Cu at room temperature. Samples were obtained from materials extruded in the direction of the extrusion axis and perpendicular to the extrusion axis.

The mechanical tests for various types of zirconium alloys were carried out in the temperature range from 400 °C to 800 °C [27]. The tests assumed 400 °C as the tensile strength test temperature. The tests were also carried out at room temperature. Fractography analysis of materials stretched at room temperature and 400 °C was carried out. Sintered and extruded materials are characterized by residual porosity, which may reduce their mechanical properties. In this work, we wanted to determine the phase composition and properties immediately after consolidation. Tensile tests of microsamples carried out in the study were aimed at determining the mechanical properties of the material after the extrusion and sintering process. The obtained material is brittle at room temperature, while at 400 °C, it shows little plasticity. The cause of the brittle fracture of the samples is the thermal stresses for the samples produced by the SPS method, and for the extruded samples, it is deformation stresses. Additional (residual) stresses can be reduced by appropriate heat treatment; however, heat treatment changes the phase composition of these materials. In this work, we wanted to determine the phase composition and properties immediately after consolidation. The tested properties were compared for materials of the same chemical composition obtained by both the SPS and the direct extrusion methods. The strength tests were carried out for samples cut in the direction of extrusion (longitudinal) and for samples cut in the direction transverse to the extrusion axis (cross) (Figure 8).

The tensile curves are similar for zirconium without additives and for Zr with 10 wt.% Cu participations, as well as for the samples extruded parallel and perpendicular to the extrusion axis. On the other hand, for 2.5 wt.% Cu, significantly higher ultimate tensile strength of 205 MPa (Table 4), approximately twice as high, was obtained for the samples cut parallel to the extrusion axis (“longitudinal”). The annealed zirconium, prepared using melting methods, is about 330 MPa [27]. For the extruded Zr without additives, for samples cut parallel to the extrusion axis, the stress is 161 MPa, while for samples cut perpendicular, it is 155 MPa.

**Figure 9:**
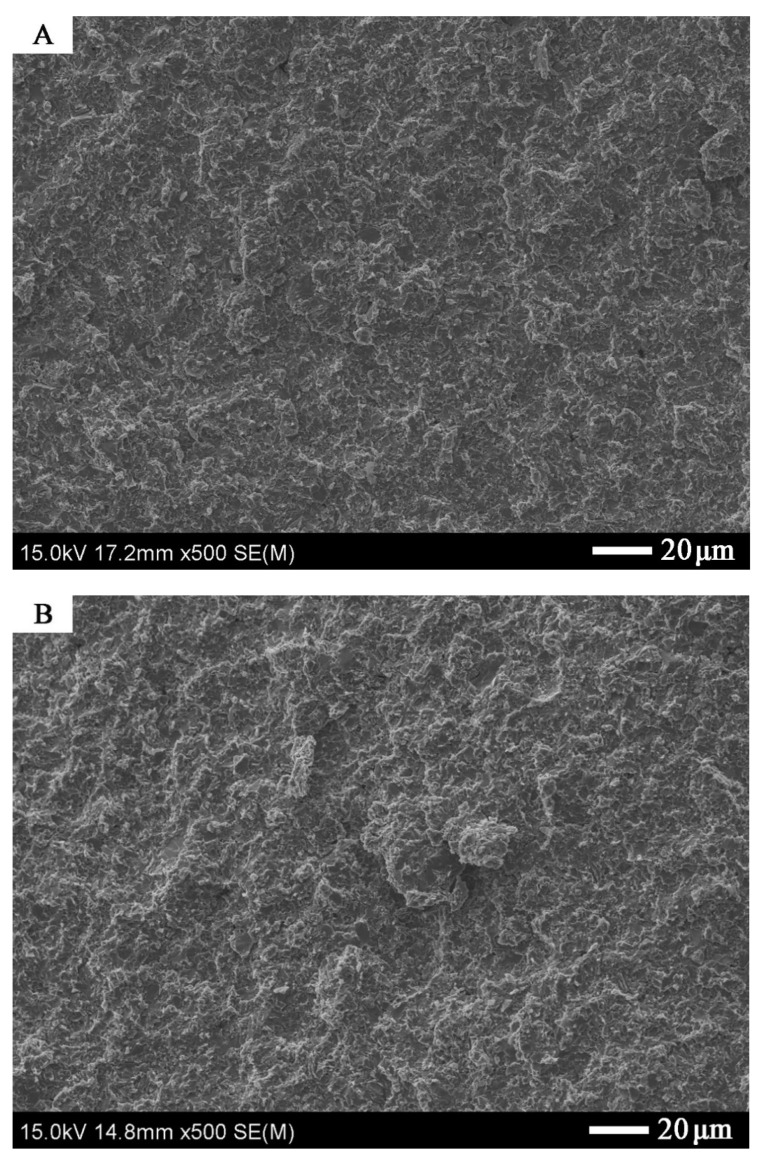
Fractography of tensile fracture surfaces for the extruded Zr-10Cu samples: (**A**) direction of the material is parallel to the extrusion axis (cut “longitudinal”); (**B**) direction of the material is perpendicular to the extrusion axis (cut “cross”).

The fractography of tensile fracture surfaces confirms a more extensive surface after the tensile test for a sample made of material extruded in the direction transverse to the extrusion axis (“cross”) (Figure 9A,B). However, for all tested samples, regardless of the direction in relation to the extrusion axis, the tensile curves correspond to the characteristics of brittle materials.

**Figure 10:**
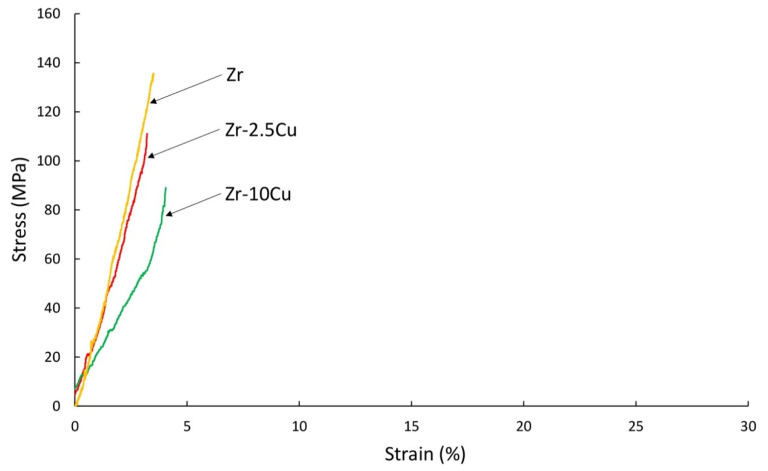
The results of the tensile test for the SPS samples of Zr-2.5Cu and Zr-10Cu at room temperature.

The tensile strength values for the sintered Zr-Cu mixtures are similar to most of the values shown in Figure 10. The tension curves are also similar to brittle materials. A decrease in strength was observed depending on the copper content for sintered materials, 101 MPa and 67, for 2.5% and 10% copper content, respectively (Table 4). For the Zr sintered material without additives, UTS is 135 MPa.

**Figure 11:**
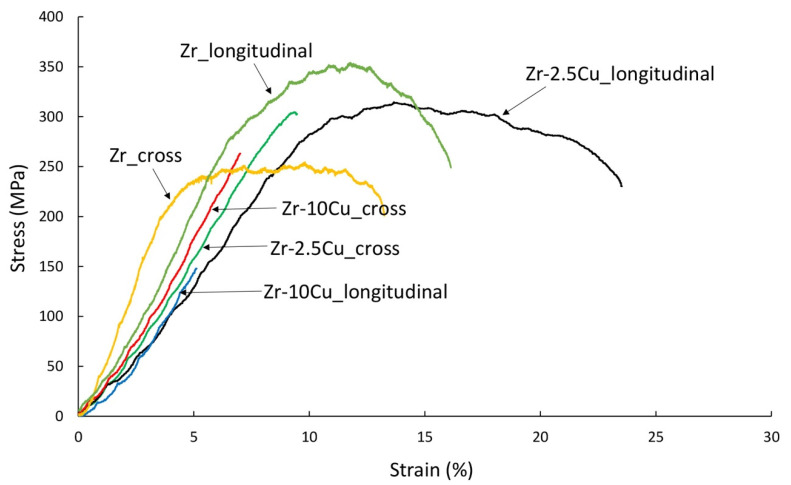
The results of the tensile test for the extruded samples of Zr-2.5Cu and Zr-10Cu at 400 °C. Samples were cut from materials obtained in the direction of the extrusion axis (“longitudinal”) and perpendicular to the extrusion axis (“cross”).

For the tensile tests shown in Figure 11 at 400 °C, the Zr-2.5Cu extruded materials exhibit plastic deformation, especially for a specimen cut parallel to the extrusion axis, “longitudinal”.

**Figure 12:**
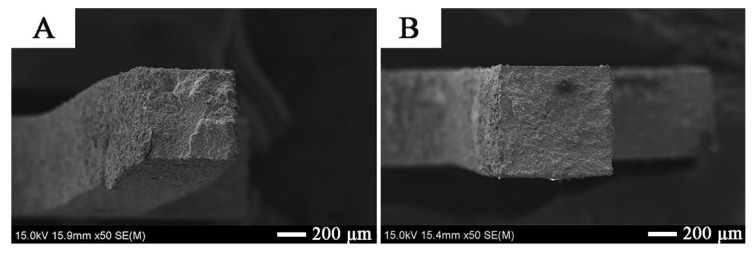
Fractography of tensile fracture surfaces for: (**A**) the extruded samples of Zr-2.5Cu and (**B**) Zr-10Cu.

The tensile deformation of the Zr-2.5Cu extruded material at 400 °C is accompanied by ductile deformation, clearly visible in Figure 12A. This type of deformation is not so clear for the Zr-10 Cu material (Figure 12B). The brittle behavior of the Zr-10Cu material is due to the greater amount of intermetallics formed during the manufacturing process.

**Figure 13:**
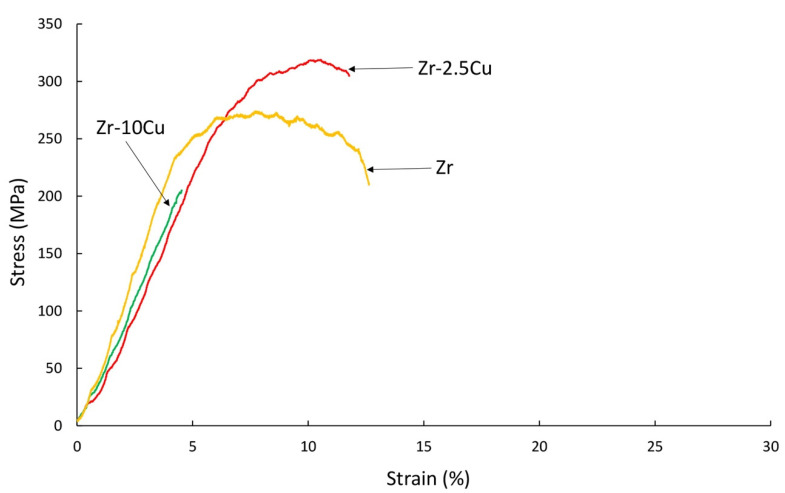
Tensile behavior of the sintered samples (SPS) of Zr-2.5Cu and Zr-10Cu at 400 °C.

The sintered materials in a tensile test at 400 °C behave similarly to extruded materials (Figure 12 and Figure 13). The material with 2.5% Cu showed the greatest plastic deformations (Figure 13). The sintered material shows less strain compared to extruded materials (Figure 12).

In Table 4, the tensile strength values for the previously described stress–strain (σ–ε) curves at room temperature and at 400 °C are presented.

Zr-xCu materials obtained from powders, extruded and sintered at room temperature, behave in a brittle manner. Ultimate tensile strength values represent 50% of the value of materials obtained by casting methods. Materials obtained from powders are characterized by residual porosity and the presence of thermal or deformation stresses. Residual stresses have a major influence on the mechanical properties of composite materials [29,30]. Heat treatment may improve the mechanical properties. Most alloys obtained by casting methods are annealed, which improves their strength properties while reducing their hardness. The obtained materials are characterized by much better properties at the temperature of 400 °C. Both for extruded and sintered materials, more favorable properties were obtained for a 2.5% Cu content. These materials are characterized by lower participation of intermetallic phases. Advantageous properties were obtained for the extruded Zr-2.5%Cu in the direction of the extrusion axis. The strain for this material at 400 °C is about 25%.

## 4. Conclusions

According to the Zr-Cu phase system, α-Zr and ZrCu_2_ should appear in this material as used consolidation parameters for Zr-2.5Cu and Zr-10Cu. For the extrusion process, ZrCu_2_ and α-Zr appear at 400 °C. For this material, a Cu_10_Zr_7_ intermetallic compound is not observed.

According to the phase equilibrium system, intermetallic Cu_10_Zr_7_ occurs for higher concentrations of Cu. Due to the presence of copper agglomerates, Cu_10_Zr_7_ is present in the composition of the sintered (SPS) Zr-Cu.

At room temperature, Zr-2.5Cu and Zr-10Cu behave like brittle materials in the tensile test, regardless of the method of obtaining materials used in the test. In the case of extruded materials, there is an anisotropy of mechanical properties related to the elongation of the grains in the direction of extrusion. The highest tensile strength value was obtained for Zr-2.5Cu obtained by the extrusion method for samples cut in the direction of extrusion.

At the temperature of 400 °C, the materials containing 2.5 wt.% Cu are characterized by a more ductile nature of the fractures. In the case of higher copper contents in the materials, there are more intermetallic phases, characterized by brittleness, which affects the brittle mechanism of fracture of the materials also at 400 °C.

The properties of materials obtained from powders are weakened by their residual porosity. The tensile strength values for the obtained materials are lower than for zirconium obtained by melting methods. The tests were carried out for non-annealing materials, which also lowers the value of mechanical properties.

## Figures and Tables

**Table 1:** Parameters describing zirconium and copper powders.

Powder	Distributor	Purity (%)	Grain Size (According to the Producer) (μm)
zirconium	BIMOTECH, Poland	99.9	>60
copper	KAMB, Poland	99.9	3.25–4.75

**Table 2:** Influence of SPS process parameters on the relative densities and hardness values of Zr, Zr-2.5Cu and Zr-10Cu.

Material	Sintering Temperature, (°C)	Duration of Sintering, (min)	Relative Density, (g/cm^3^)	Vickers Hardness, HV2	Standard Deviation
Zr *	1200	1	98.00	409	±3.0
Zr-2.5Cu	800	1	97.08	364	±9.3
Zr-2.5Cu	850	1	98.08	464	±9.4
Zr-2.5Cu	900	1	98.70	476	±2.1
Zr-10Cu	800	1	95.74	421	±6.7
Zr-10Cu	850	1	98.53	479	±7.7
Zr-10Cu	900	1	99.01	467	±6.4

* Zirconium sintering parameters without additives were optimized in previous studies [5].

**Table 3:** Influence of the extrusion process parameters on the relative densities and hardness values of Zr, Zr2.5Cu and Zr10Cu.

Material	Temperature of Extrusion (°C)	Extrusion Speed (Punch Displacement) (mm/s)	Relative Density (%)	Vickers Hardness * HV2 “Across”	Standard Deviation	Vickers Hardness * HV2 “Longitudinal”	Standard Deviation
Zr	400	3.7	97.05	407	±5.3	402	±6.55
Zr-2.5Cu	400	3.7	97.31	452	±7.2	450	±5.3
Zr-10Cu	400	3.7	96.47	455	±8.2	460	±6.6

* Samples were cut from materials extruded in the direction of the extrusion axis (“longitudinal”) and perpendicular to the extrusion axis (“cross”).

**Table 4:** Ultimate tensile strengths and other mechanical parameters for extruded and sintered Zr-xCu materials.

Material/Manufacturing Process	Test Temperature, (°C)	The Direction of Samples	Ultimate Tensile Strength *, (MPa)
Zr/extrusion	RT	Longitudinal	161
Zr/extrusion	RT	Cross	155
Zr/SPS	RT	–	135
Zr/extrusion	400	Longitudinal	356
Zr/extrusion	400	Cross	293
Zr/SPS	400	–	285
Zr-2.5Cu/extrusion	RT	Longitudinal	205
Zr-2.5Cu/extrusion	RT	Cross	108
Zr-2.5Cu/SPS	RT	–	101
Zr-2.5Cu/extrusion	400	Longitudinal	325
Zr-2.5Cu/extrusion	400	Cross	324
Zr-2.5Cu/SPS	400	–	329
Zr-10Cu/extrusion	RT	Longitudinal	92
Zr-10Cu/extrusion	RT	Cross	107

* The value of ultimate tensile strength is the mean of the measurements for the five samples.

## Data Availability

The data presented in this study are available on request from the corresponding author.
